# The 17β-estradiol induced upregulation of the adhesion G-protein coupled receptor (ADGRG7) is modulated by ESRα and SP1 complex

**DOI:** 10.1242/bio.037390

**Published:** 2018-12-31

**Authors:** Amani Hassan, Edward T. Bagu, Mathieu Levesque, Shunmoogum A. Patten, Samira Benhadjeba, Lydia Edjekouane, Isabelle Villemure, André Tremblay, Florina Moldovan

**Affiliations:** 1 CHU Sainte Justine Research Center, Montréal H3T 1C5, Canada; 2 Department of Basic Biomedical Sciences, Sanford Medical School, University of South Dakota, Vermillion, SD 57069, USA; 3 INRS-Institute Armand-Frappier, Laval H7V 1B7, Canada; 4 Department of Mechanical Engineering, Ecole Polytechnique de Montréal, Montréal H3T 1J4, Canada; 5 Department of Stomatology, Faculty of Dentistry, Université de Montréal, Montréal H3C 3J7, Canada

**Keywords:** AIS, Adhesion G-protein coupled receptor, ADGRG7, GPR128, Estrogen, Estradiol (E2), Gene regulation, Osteoblasts

## Abstract

The physiological role and the regulation of ADGRG7 are not yet elucidated. The functional involvement of this receptor was linked with different physiological process such as reduced body weight, gastrointestinal function and recently, a gene variant in ADGRG7 was observed in patients with adolescent idiopathic scoliosis. Here, we identify the *ADGRG7* as an estrogen-responsive gene under the regulation of estrogen receptor ERα in scoliotic osteoblasts and other cells lines. We found that *ADGRG7* expression was upregulated in response to estrogen (E2) in adolescent idiopathic scoliosis (AIS) cells. *ADGRG7* promoter studies indicate the presence of an ERα response half site in close vicinity of a specificity protein 1 (SP1) binding site. Mutation of the SP1 site completely abrogated the response to E2, indicating its essential requirement. ChIP confirmed the binding of SP1 and ERα to the *ADGRG7* promoter. Our results identify the *ADGRG7* gene as an estrogen-responsive gene under the control of ERα and SP1 tethered actions, suggesting a possible role of estrogens in the regulation of *ADGRG7*.

This article has an associated First Person interview with the first author of the paper.

## Abstract (precis)

**Summary:** Estrogen plays a significant role in AIS and studying the regulation of ADGRG7 by E2 in AIS cells is essential for understanding molecular mechanisms underlying AIS pathogenesis.

## INTRODUCTION

Adolescent idiopathic scoliosis (AIS) is a complex three-dimensional deformity of the spine that mostly occurs during late childhood or puberty (Konieczny et al., 2013). Severe forms of AIS are more common in girls compared to boys (Cheng et al., 2015). The difference between girls and boys, as well as the etiology of AIS, are still unclear. Several studies suggest that AIS could be an endocrinal disease and that various hormones, especially estrogens, have a role in its onset, development and spinal curve progression (Barrios et al., 2011). Lower peak bone mass and osteopenia at puberty have been reported in 27%–38% of AIS patients, suggesting that AIS may be correlated with hormonal disturbances involving estrogen, melatonin and leptin (Ishida et al., 2015). Estrogens (including estradiol) (E2) and estrogen receptors (ERs), including the ERα and ERβ isoforms, are suspected of influencing AIS severity and delayed puberty which has been directly associated with a higher prevalence of AIS in girls than in boys with an incidence ratio of 7.1:1 (Konieczny et al., 2013). Indeed, several ER polymorphisms were found in AIS (Stavrou et al., 2002), but the predisposition to and severity of AIS was not clearly demonstrated (Janusz et al., 2014). E2 participation in puberty, spinal growth and bone metabolism is an important factor to consider in AIS. Until now, it was not clear how E2 could affect the initiation or progression of AIS. However, estrogens interact with many physiopathological factors (including neuroendocrine, neurological, muscular, biochemical and structural) relevant to the etiology of scoliosis, and there is interdependence between the concentration of E2 and development of scoliosis (Leboeuf et al., 2009).

Estradiol is the major hormonal regulator of puberty and bone metabolism and acts through genomic and non-genomic pathways. The genomic effects of E2 are exerted by its binding to the ER in the cytoplasm. This is followed by the translocation to the nucleus and binding of the complex to target genes. In addition to the direct genomic signaling through ER (Dahlman-Wright et al., 2006; Prossnitz et al., 2008), ERs can also mediate their transcriptional potential through tethered interaction with other transcription factors, such as specificity protein 1 (SP1) and activator protein (AP1). In these cases, most estrogen-responsive genes are devoid of estrogen response elements (ERE) (Björnström and Sjöberg, 2005), suggesting enhanced recruitment of ER ligands to target promoters through protein–protein interaction such as SP1 (Safe, 2001). SP1 is a ubiquitously expressed transcription factor that binds and acts through GC-rich elements to regulate gene expression in mammalian cells (Li et al., 2007; Keay and Thornton, 2009).

The adhesion G protein-coupled receptor 7 (ADGRG7), previously known as the G protein-coupled receptor 128 (GPR128), is a membrane-bound protein encoded by *ADGRG7* (Fredriksson et al., 2002; Bjarnadóttir et al., 2004; Arac et al., 2012a). In humans and mice, the *ADGRG7* gene is on chromosome 3q12.2 and 16; 16 C1.1, respectively (Fredriksson et al., 2002; Bjarnadóttir et al., 2004; Arac et al., 2012a). ADGRG7 is an orphan receptor that belongs to the family of proteins that consists of over 33 homologous proteins (Bjarnadóttir et al., 2004; Yona et al., 2008; Yona and Stacey, 2010). Like most members of ADGRG family, the extracellular region often a N-terminal protein module is extended and linked to a transmembrane (TM) 7 region via the GPCR-autoproteolysis inducing (GAIN) domain (Arac et al., 2012a). *ADGRG7,* which is phylogenetically related to *ADGRG2* and *ADGRG1,* lacks the conserved N-termini domains present in other GPCRs (Foord et al., 2002; Bjarnadóttir et al., 2004; Huang et al., 2012). ADGRG7 was shown to be expressed in the mucosa of the intestine restricted to the epithelial cells (Badiali et al., 2012; Ni et al., 2014).

The physiological role of ADGRG7 remains mostly unclear. The GPCR family of proteins are mainly involved in cellular adhesion, migration, cell–cell and cell–matrix interactions (Yona et al., 2008). In mice, targeted deletions of the *ADGRG7* gene reduced weight gain and increased the frequency of peristaltic contractions of the small intestine, suggesting a role in intestinal absorption of nutrients (Badiali et al., 2012). An important paralog of this gene is *ADGRG6*, which is suggested as playing a role in musculoskeletal disorders such as AIS and pectus excavatum (PE) (Kou et al., 2013; Karner et al., 2015).

In humans, *ADGRG6* gene variants were first associated with AIS in the Japanese population and then a single nucleotide polymorphism (SNP) in *ADGRG6* gene (rs657050) was replicated in Han Chinese and European-ancestry AIS population. In zebrafish, the *adgrg6* knockdown causes delayed ossification of the developing spine (Kou et al., 2013) and in a mouse model, the loss of *Adgrg6* in osteochondroprogenitor cells affects spinal column development and intervertebral disk morphogenesis (Karner et al., 2015).

*ADGRG7* was also suggested among the genetic causes or genetic contributors for the pathogenesis of AIS. The *ADGRG7* gene maps on the chromosome 3q12.1. Through linkage analysis in multigenerational AIS families with dominant inheritance this locus was reported as one of the two locations containing the gene for AIS (Edery et al., 2011). Our recent study (Patten et al., 2015) identified by exome sequencing two candidate gene variants (SNV) among the novel or rare [minor allele frequency (MAF) <5%] variants: one in *ADGRG7* and the other in *POC5* (Patten et al., 2015)*.* The *ADGRG7* SNV (1274A>G) did not perfectly co-segregate with AIS in all the members of this multigenerational AIS family; consequently, the *ADGRG7* gene was concluded as a contributory/modifier gene in the pathogenesis of AIS. Based on these findings, and because *ADGRG7* is closely related to the *ADGRG6* (gene implicated in AIS), we hypothesized that *ADGRG7* is regulated by E2 and consequently can contribute to the cellular events in AIS.

To examine how *ADGRG7* is regulated at the transcriptional and protein level by E2, we conducted promoter and deletion analysis. We also conducted gene and protein expression study in human osteoblasts, Huh7 and MCF7 cells. Human *ADGRG7* gene was cloned and analyzed for functional *cis-*elements mediating the effects of E2. Deletion analysis of the promoter identified the SP1 site required for both basal activity and hormone-induced activation. Chromatin immunoprecipitation (ChIP) assay confirmed that SP1/ERα binds to *ADGRG7* promoter. Our study suggests that the regulation of ADGRG7 expression by E2 is due to the association of ERα and SP1 proteins to *ADGRG7* promoter.

## RESULTS

### Gene expression profile of ADGRG7 and SP1 in multiple human tissues

The ADGRG7 has been poorly characterized in terms of function and tissue expression. We therefore analyzed the expression levels of ADGRG7 and SP1 in different tissues (Fig. S1) using the Gene Expression Omnibus (GEO) database at the US National Center for Biotechnology Information (NCBI). We found that ADGRG7 was highly expressed in the small intestine, as previously reported (Badiali et al., 2012). However, unlike in mice, ADGRG7 expression was not selective for the intestine: ADGRG7 was also expressed in the liver, pancreas and placenta. The SP1 transcription factor was highly expressed in the pancreas with wide expression in all tissues tested except for the skin. Interestingly, ADGRG7 and SP1 were also expressed in the bone; this suggests a wider function than expected and an undetermined role of ADGRG7 in bone.

### Dose-dependent differential upregulation of *ADGRG7* by 17β-estradiol in normal osteoblasts (NOB) and AIS cells

**Fig. 1.**
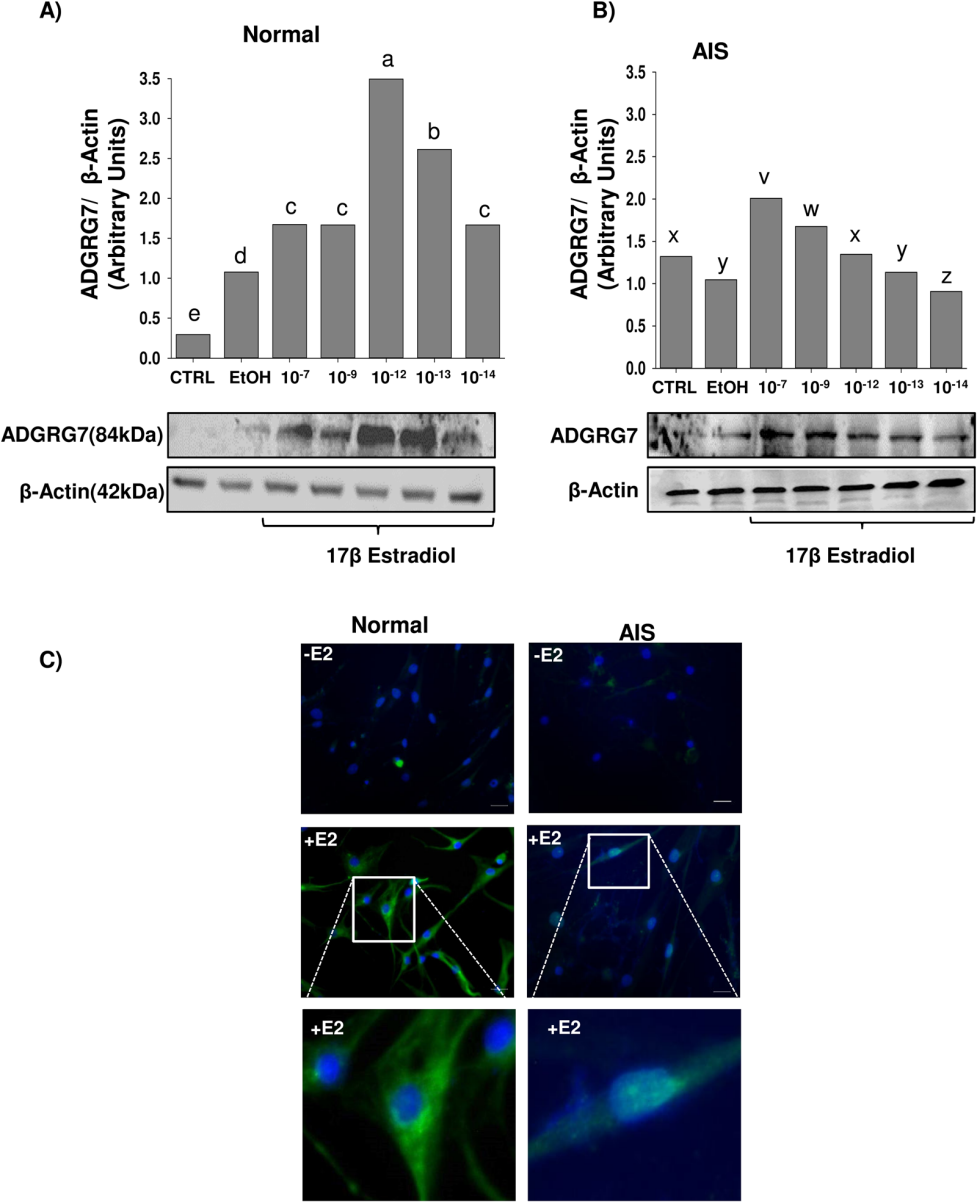
**Upregulation of ADGRG7 expression in NOB and AIS osteoblasts by 17β-Estradiol (E2).** (A,B) NOB and AIS cells were treated with different doses of E2 (10^−7^, 10^−9^, 10^−12^, 10^−13^ and 10^−14^ M) E2 for 24 h followed by analysis of whole cell extract by western blotting using anti-ADGRG7 antibody. Anti-β-actin antibody was used as a loading control. Protein quantification was performed using ImageJ. Different letters indicate paired means that are statistically different (*P*<0.05). For variables with the same letter, the difference is not statistically significant. Fisher's LSD test was used for statistical analysis. (C) Immunolocalization of ADGRG7 in NOB and AIS cells cultured without E2 (−E2) or with E2 (+E2) overnight in CS-FBS medium. Cells were cultured on Labteck (as described in the Materials and Methods), fixed, permeabilized and analyzed for ADGRG7 positive immunostaining. Note the cytoplasmic staining of ADGRG7 in cells cultured overnight in defined medium and the increase in immunostaining after 24 h treatment with E2. Nucleus is stained with DAPI (blue).

To characterize changes in gene expression in response to E2 treatment in normal control osteoblasts (NOB) and AIS osteoblasts, ADGRG7 was differentially regulated by E2 treatment in NOB and AIS cells. E2 upregulated ADGRG7 protein levels at 10^−7^ M E2 in normal osteoblasts with maximal response at 10^−12^ and 10^−13^ M (Fig. 1A). AIS cells also responded to E2 at lower levels than normal osteoblasts (Fig. 1B). In both NOB and AIS cells, there was a dose-dependent increase in steady-state levels of ADGRG7. The densitometry signals for the E2-treated control NOB and AIS cell samples were normalized to the β-actin signal and yielded normalized densitometry ratios. The data for the normalized ratios for the specific protein ADGRG7 are summarized in (Fig. 1A,B).

To test whether E2 affects ADGRG7 protein localization, we performed applied fluorescence imaging technique using ADGRG7 antibody. We observed low staining in untreated cells, enhanced staining after treatment with E2 in NOB cells (Fig. 1C) and low staining in AIS cells. These results are consistent with the results of upregulation of ADGRG7 by E2 in western blot (Fig. 1A,B).

The ability of ERα and SP1 to interact with each other and DNA along with the differential expressions of ERα in different normal and AIS patient cells, led us to examine the expression levels of SP1 and ERα in NOB and AIS cells. We observed by western blot differential expression patterns of both proteins. SP1 and ERα were expressed at higher levels in NOB compared to AIS cells (Fig. S2).

These studies reveal that ADGRG7 is a cytoplasmic protein regulated by E2 in NOB osteoblasts and at lower levels in AIS cells (Fig. 1A–C). SP1 and ERα protein levels were also higher in NOB cells than AIS cells (Fig. S2).

### Transcriptional activation of *ADGRG7* and deletion analysis of the *ADGRG7* gene promoter

**Fig. 2.**
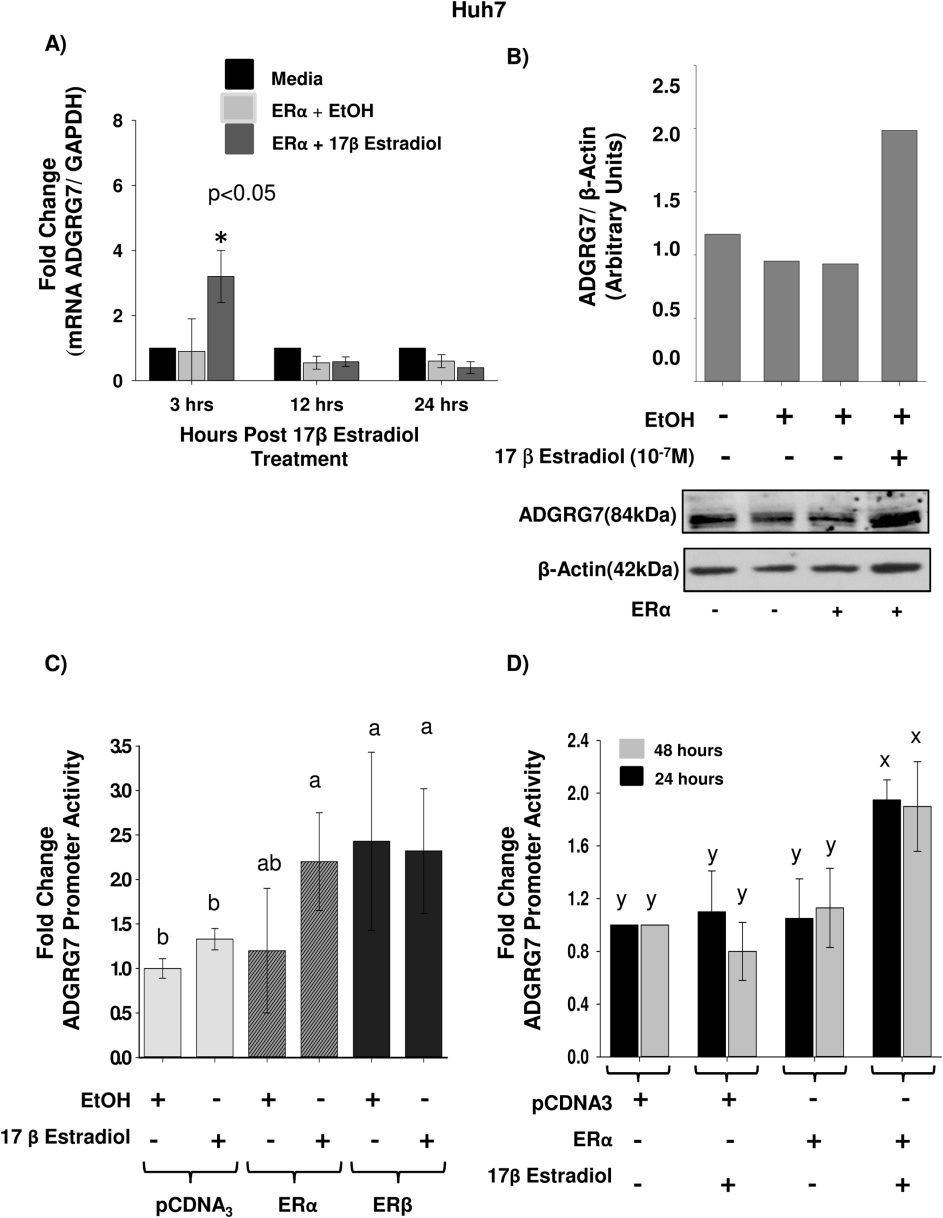
**ADGRG7 expression is upregulated by E2 in Huh-7 cells.** (A) E2 modulates ADGRG7 gene expression. Human hepatocellular carcinoma cells, Huh7, were transiently transfected with either an expression plasmid vector that encodes the full length wild-type estrogen receptor alpha (ERα) or with the empty expression vector back bone (pCDNA3 plasmid vector). The cells were then treated with E2 (10^−7^ M), and the control cells transfected with the empty pCDNA3 vector were treated with the vehicle (ethanol). The Huh7 cells were treated with E2 for 3 h, 12 h and 24 h. ADGRG7 expression was measured by qPCR. The gene expression levels of ADGRG7 in Huh7 cells following treatment with E2 were determined relative to that of a housekeeping gene, GAPDH and the fold change was calculated relative to the controls for each time point. The controls at each time point were Huh7 cells transfected with pCDNA3 and treated with vehicle (ethanol) for 3 h, 12 h and 24 h. *P<0.05 statistically significant differences between E2 and medium or EtOH treated samples. (B) Western blot analysis of ADGRG7. Immunoblot analysis for ADGRG7 was performed following 24 h post treatment with E2 (10^−7^ M) or ethanol. The expression of ADGRG7 was determined relative to the housekeeping gene β-actin that was used as a loading control. The mean relative protein expression levels were quantified by densitometry analysis of immunoblots using ImageJ. (C) ERα but not ERβ mediates ADGRG7 upregulation in Huh-7 cells. The ADGRG7 promoter activity (−1285/+112; 500 ng/well) in Huh-7 cells was determined following co-transfection with an expression vector that encodes for the full length wild type estrogen receptor protein (ERα or ERβ) and treatment with E2 (10^−7^ M) for 24 h. (D) Co-transfection with ERα, and then treatment with E2 (10^−7^ M) for 24 h or 48 h. Controls in each case were transfected with the empty pCDNA3 vector (Invitrogen) and then treated with the vehicle (ethanol). Results are shown as fold activation over control (mean±S.E.M.). In C and D, the different letters are used to indicate paired means that are statistically different (*P*<0.05). For variables with different letters, the difference was statistically significant (*P*<0.05). For variables with similar letters, the difference is not statistically significant. Fisher's LSD test was used for statistical analysis.

In order to address the regulation of *ADGRG7* gene expression in response to E2, we treated Huh-7 cells at three different time points (3 h, 12 h and 24 h). Since Huh-7 cells have low levels of expression of ER, we overexpressed the estrogen receptor-construct or pcDNA3 mock vector as control. Cells were either non-treated or treated with ethanol or E2. We found that *ADGRG7* has maximal response to E2 after 3 h treatment. From 12 h and 24 h, we did not find differences between treated and untreated samples. This suggests an early response gene to E2 (Fig. 2A). At the protein level, ADGRG7 was upregulated with a 10^−7^ M treatment of E2 for 24 h (Fig. 2B). We assessed by luciferase assay if the regulation of *ADGRG7* is through ERα or ERβ in Huh-7 cells, and we found that *ADGRG7* promoter (−2259/+55) is upregulated by E2 in the presence of ERα but not ERβ (Fig. 2C). Finally, we observed similar responses to E2 at 24 h and 48 h post-treatment (Fig. 2D). *ADGRG7* is upregulated by E2 through ERα at 3 h and at lower levels at 24 h and 48 h post-treatments with E2.

### Deletion analysis of the proximal region of the *ADGRG7* promoter

**Fig. 3.**
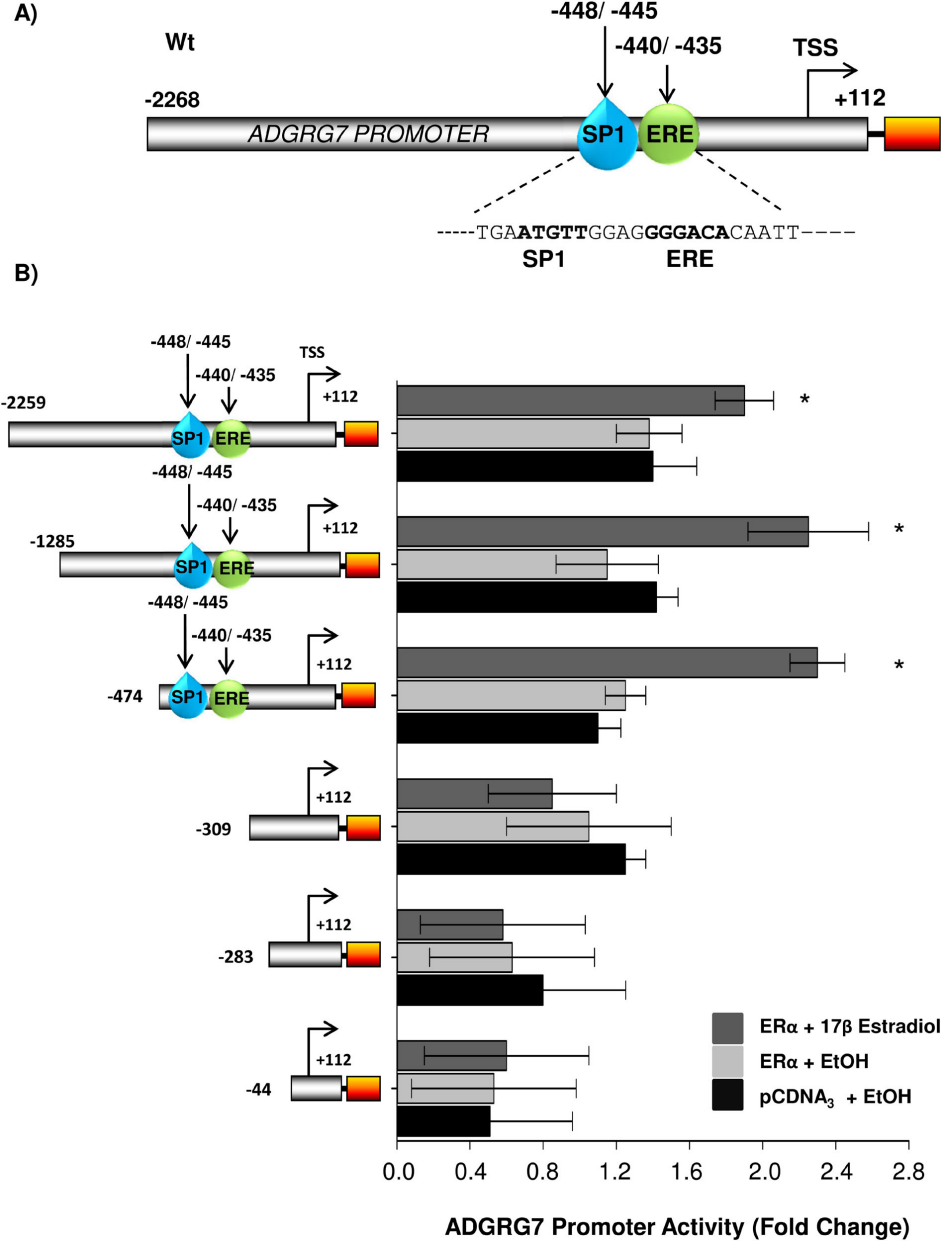
**Regulation of the proximal promoter activity of ADGRG7 by ERα.** (A) Schema representing the ADGRG7 full length promoter with SP1 and ERE1/2 as well as elements and position with respect to transcription start site (TSS). (B) Huh-7 cells were transfected with ERα in the presence of luciferase reporter construct containing the full- length promoter (−2259/+55) of ADGRG7 and its serial deletions constructs (−1285/+55, −474/+55; −309/+55 and −283/+55 and −44/+55). Cells were then treated or not with 10^−7^ M E2 for 24 h. The arrow pointing to the right represents the transcription start site (TSS). Luciferase values were normalized to Renilla luciferase activity and expressed as relative fold response compared to vehicle-treated mock transfected cells set for each experiment at 1. Mean values (S.E.M.) of at least three separate transfections in triplicates are shown. Statistical comparisons were performed between E2 and vehicle treated samples. *P<0.05 was considered statistically significant from the control (pCDNA3/EtOH treated) of the same construct. Newman-Keuls test was used.

To determine the responsive region responsible for the estrogenic upregulation of the *ADGRG7* gene, we performed reporter gene assay in Huh-7 cells using a portion of 2.2 kb proximal to the transcription start site (TSS) of the *ADGRG7* promoter. A schematic of the proximal promoter of the *ADGRG7* gene shows the presence of several putative ERE binding elements (Fig. 3A). The co-transfection of hERα and various truncations of the promoter series, of 5′- deletion constructs including the −2259 to +112, −1285 to +112, −474 to +112, −309 to +112, −283 to +112 and −44 to +112 regions of the *ADGRG7* gene promoter were used in transient transfection studies to identify specific E2-responsive elements within this region of the promoter (Fig. 3B). We found that the estrogenic induction was retained with the −474 construct and then lost with the −309 construct, suggesting that this region is essential for the E2 response of the promoter (Fig. 3B). Further deletions were not inducible by E2. These results indicate that (i) the stimulation of the promoter by E2 requires the presence of its receptor and (ii) the region between 474 and 309 bp encompasses potential regulatory elements sufficient to confer regulation by E2.

### An essential role for SP1, ERE1/2 motif in signaling estrogen regulation of the *ADGRG7* promoter

**Fig. 4.**
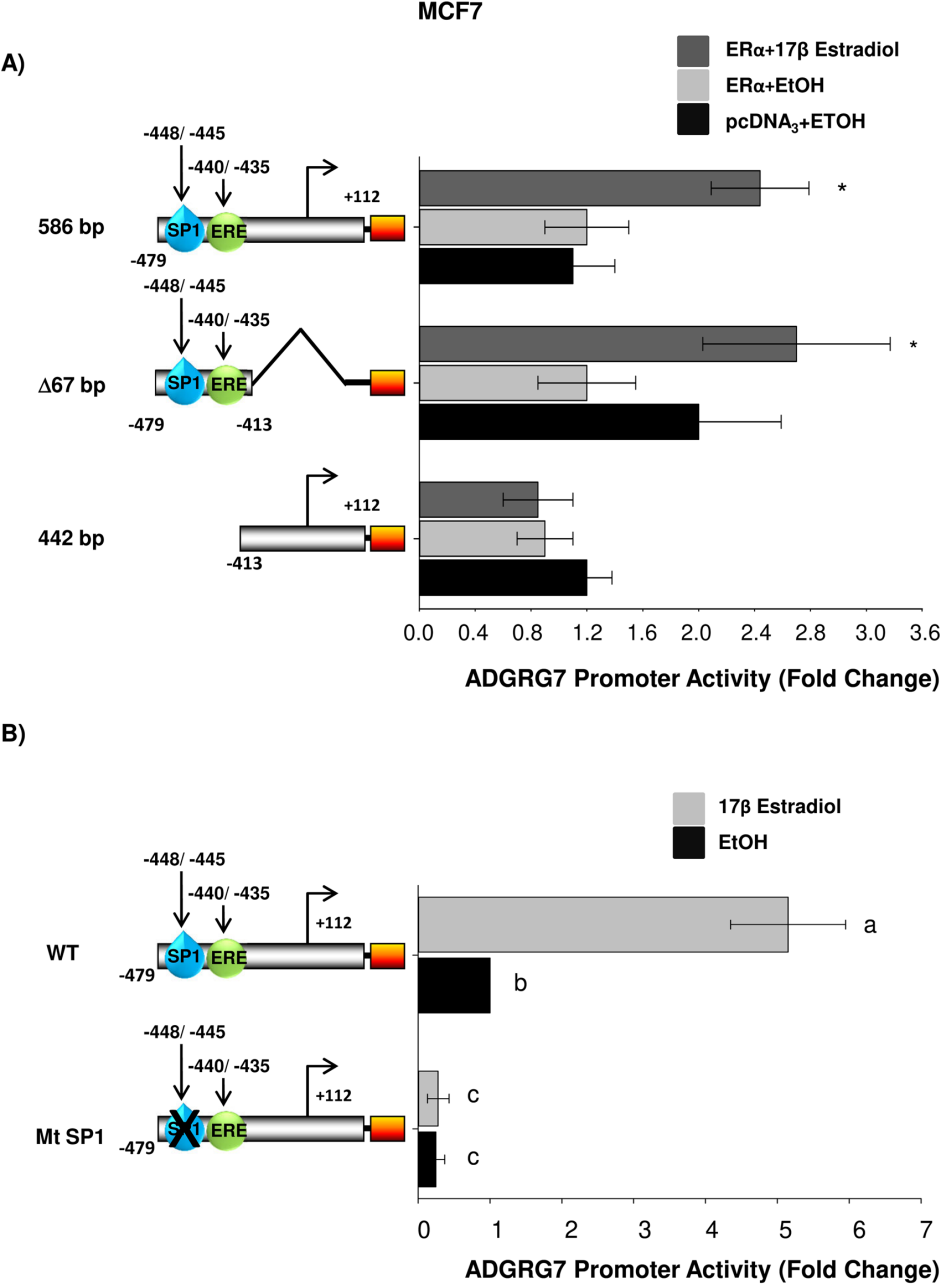
**Localization of a functional region within ADGRG7 promoter is important for activation of ADGRG7 transcription.** (A) ADGRG7 wild-type (586 bp) truncated (Δ67 bp and 44 bp) promoter activity was determined in MCF-7 cells treated with 10^−7^ M E2 or vehicle for 24 h. Cells were analyzed for luciferase activity. *P<0.05 statistically significant difference between vehicle and E2 treated samples. (B) ADGRG7 wild-type and mutant SP1 promoters were analyzed for luciferase activity as in A. The different letters are used to indicate paired means that are statistically different (*P*<0.05). Paired means (SP1 mutant with E2 or EtOH treatments) that are non-significantly different are indicated by the same letter. Fisher's LSD test was used for statistical analysis.

Deletion analysis of the SP1×ERE1/2 of the *ADGRG7* promoter was carried out to further define their role in functional interactions with ERα/SP1 (Fig. 4A). The 586 bp and Δ67 bp were E2-responsive, and deletion analysis was used to determine contributions of the upstream SP1×ERE1/2 binding sites. E2 did not significantly induce luciferase activity in cells transfected with 442 bp fragment. To provide experimental evidence that the activation of *ADGRG7* by E2 is mediated by an Sp1-binding element, we transiently transfected MCF7 cells, which are ERα positive, with a luciferase reporter gene driven by a promoter carrying SP1mut. Relative luciferase activity data showed the absence of response to E2 treatment (Fig. 4B). Results obtained for these deletion/mutant constructs (Fig. 4A,B) indicate that the SP1×ERE1/2 in the Δ67 region of the *ADGRG7* gene is important for hormonal activation by ERα/SP1. These studies support a role for the SP1×ERE1/2 site in mediating estrogen responsiveness of the human *ADGRG7* gene.

### Physical and functional interactions of SP1 and ERα

**Fig. 5.**
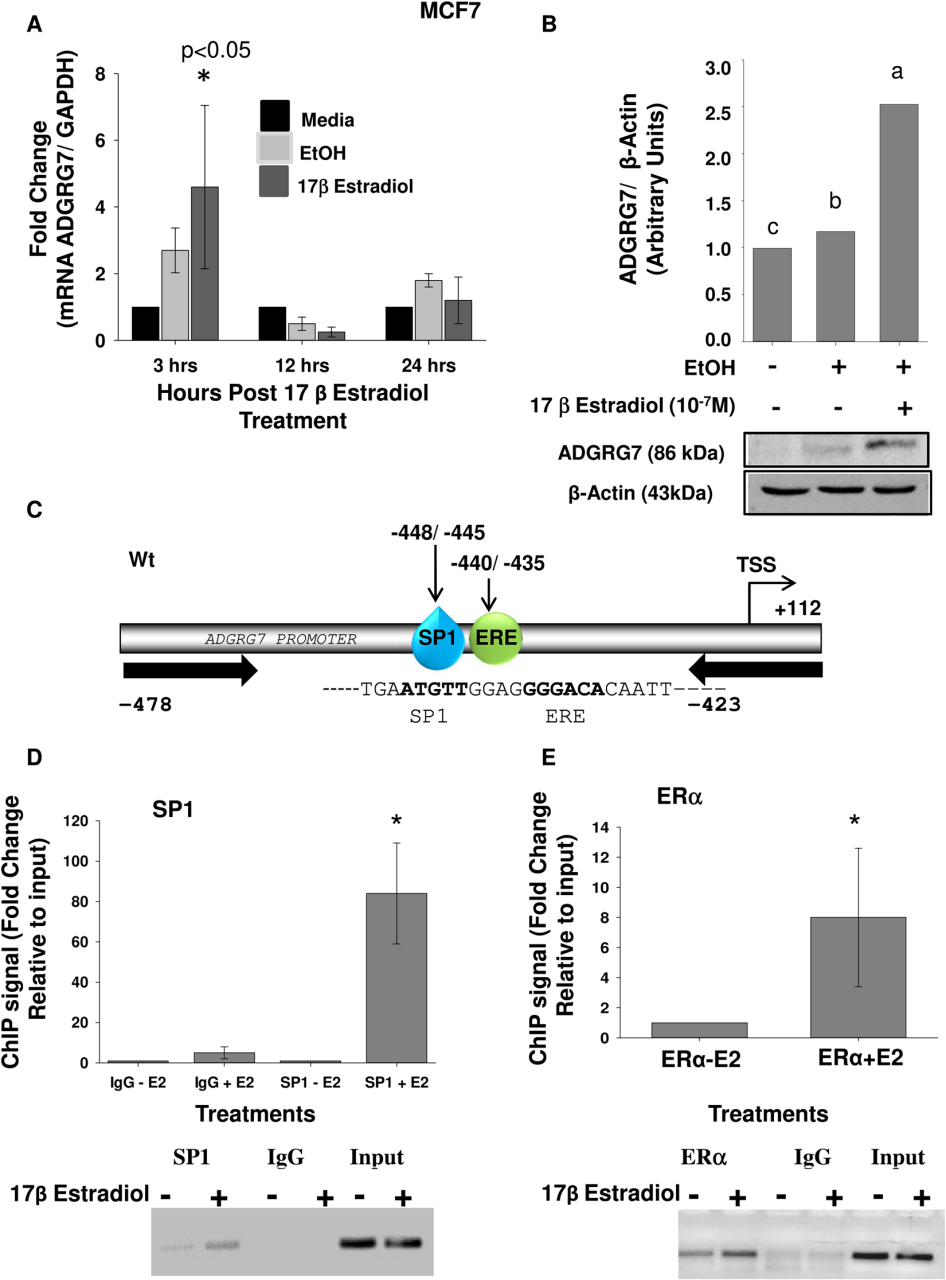
**Specificity protein 1 (SP1) and ERα bind to the ADGRG7 proximal promoter in MCF-7 cells in the presence of E2.** (A) qPCR expression of ADGRG7 in MCF7 cells in response to E2 at three different time points (3 h, 12 h and 24 h). Statistical comparison was performed between pCDNA3 (EtOH) and E2-treated samples of the same construct. *P<0.05. (B) Immunoblot analysis for ADGRG7 in control and cells treated E2 for 24 h. The different letters are used to indicate paired means that are statistically different (*P*<0.05). In the representative immunoblots, the protein was detectable at the expected size of 85 kDa for ADGRG7. Anti-β-actin antibody was used as a loading control. Protein quantification was performed using ImageJ. (C) Schematic representation of Δ67 bp fragment of the ADGRG7 showing approximate locations of ERE1/2 half-sites and SP1 binding sites. (D) SP1 and ERα binding to the ADGRG7 promoter. ChIP-qPCR validation of SP1×ERE1/2 binding sites identified from ChIP-seq analysis in vicinity of the ADGRG7. MCF-7 cells were treated with 10^−7^ M E2 for 1 h. Results represent fold enrichment values obtained by comparing CT values of ChIP samples to the CT values of input. The qPCR products were migrated on gel to confirm the correct product size. *P<0.05 indicate difference between vehicle and E2-treated cells. Fisher's LSD test was used for statistical analysis.

We studied the gene expression patterns induced in MCF7 ERα-positive, estrogen-dependent breast cancer cell line, grown in steroid-depleted medium or in the presence of E2. As observed in Huh-7 cells, E2-induced mRNA expression occurred at 3 h and this response returned to basal levels after 12 h and 24 h (Fig. 5A). ADGRG7 protein levels were upregulated 2.5-fold in MCF7 cells (Fig. 5B).

We next determined the interactions of ERα and SP1 proteins with the ADGRG7 gene promoter in MCF7 cells treated with 10^−7^ M E2 for 1 h using a ChIP assay. The recruitment of ERα and SP1 proteins with the proximal region of the *ADGRG7* promoter (−448 to −435) was investigated and the results indicated that both ERα and SP1 antibodies immunoprecipitate this region of *ADGRG7* promoter as determined by qPCR (Fig. 5C).

We showed that *ADGRG7* is also upregulated by E2 in MCF7 cells. Through a ChIP experiment, we confirmed the binding of SP1 and ERα to *ADGRG7* promoter after stimulation with E2 for 1 h.

### ERα antagonists 4-hydroxy tamoxifen (4-OHT) and fulvestrant (ICI-182, 780) reverse the upregulatory effects of E2 on *ADGRG7* expression and promoter activity

**Fig. 6.**
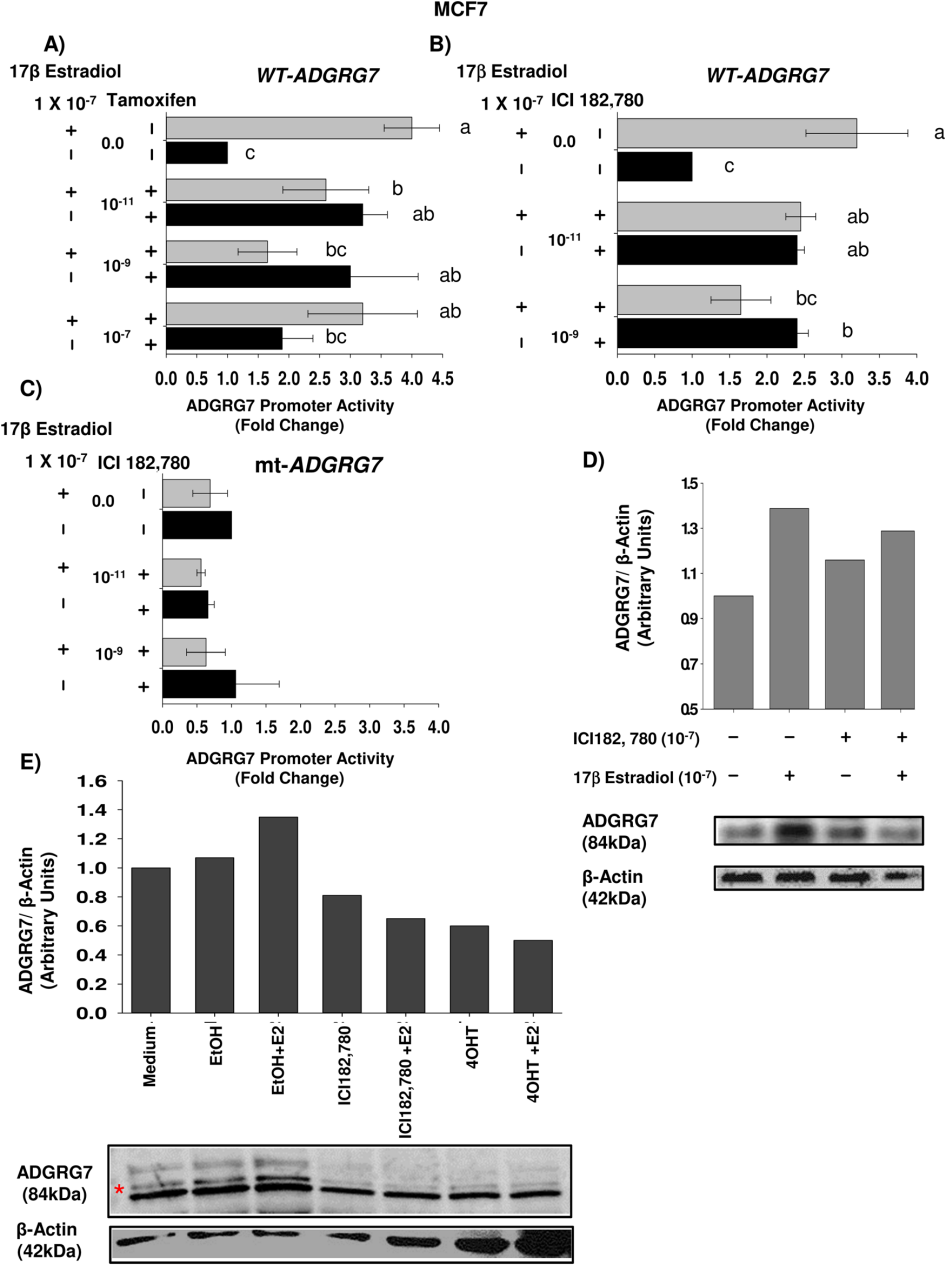
**Effects of E2, ICI-182, 780 and 4-OHT on the induction of SP1- and ERα-driven reporter activities of ADGRG7 in MCF7 cells.** (A) Dose response of ADGRG7 promoter to 4-OHT. Cells were transiently transfected with 479 bp luciferase reporter plasmid. After 24 h of transfection, cells were treated with vehicle only, 10^−7^ M estradiol (E2), and different doses 10^−7^, 10^−9^, and 10^−11^ M 4-OHT, or 10^−7^ M E2+4-OHT (E2+4-OHT). After 24 h, cells were harvested and assayed for luciferase activity. Luciferase values were corrected for transfection efficiency by measuring the Renilla luciferase activity. Three individual experiments were performed. Data are presented as mean and standard mean error (error bars). One-way ANOVA with Tukey B post hoc analysis was applied to determine significance among different treatment groups in this experiment. Different means are designated by different letters. ab indicates that the difference between the mean of treatment with different doses of 4-OHT (10^−11^ and 10^−9^ M) or 10^−7^ M 4-OHT+E2 is not significantly different from a (E2) and b (E2+10^−11^ M 4-OHT). bc indicates that the mean of the treatment with 10^−7^ M 4-OHT or 10^−9^ M 4-OHT+E2 is not statistically significant from c (EtOH treated) and b (10^−11^ M 4-OHT+E2). Newman-Keuls test was used. (B) Dose response of ICI-182, 780 on ADGRG7 promoter regulation. Transfection experiments were performed same as A. After 24 h of transfection, cells were treated with vehicle, 10^−7^ M estradiol (E2), 10^−9^ and 10^−11^ M ICI-182, 780, or 10^−7^ M E2+ICI-182, 780 (E2+ICI). Letters indicate statistically significant difference of the means of E2, vehicle and 10^−9^ M ICI-182, 780. ab indicates that the mean of the treatments 10^−11^ M ICI-182, 780 and 10^−11^ M ICI-182, 780+E2 is not different from E2 and 10^−9^ M ICI-182, 780. bc indicates that the mean of 10^−9^ M ICI-182, 780+E2 is similar to b (10^−9^ M ICI-182, 780) and c (EtOH treated). The different letters used in A and B indicate means that are statistically different (*P*<0.05). Newman-Keuls test was used. (C) Effects of 10^−7^ M E2 and 10^−7^ M ICI-182, 780 on SP1 mutant reporter construct. Transfections were performed as in A and B. No difference was obtained between the means of the E2, vehicle and ICI 182, 780 of the SP1mut construct. Newman-Keuls test was used. (D) Effect of E2 or ICI-182, 780 on ADGRG7 protein in MCF-7 cells. Cell lysates were prepared at the indicated treatment times with either 10^–7^ M E2 or 10^–7^ M ICI-182, 780. The ADGRG7 protein was detected using a primary polyclonal rabbit antibody and the immunocomplex was visualized using the enhanced chemiluminescence (ECL) detection system (Amersham). (E) Western blot analysis of E2, ICI-182, 780 and 4-OHT effects on the expression of ADGRG7 protein. Protein expression was analyzed in cells untreated or treated for 24 h with 10^−7^ M E2 or 10^−7^ M ICI-182, 780 or 10^−7^ M 4-OHT. Expression of β-actin was used as control.

We tested the effect of 4-OHT, which is considered a context-dependent mixed agonist/antagonist of ERα (Fig. 6A). Interestingly, while 4-OHT was able to block the E2 activation of the *ADGRG7* promoter thus acting as an ERα antagonist, when used in absence of E2, 4-OHT induced ADGRG7 promoter activity in MCF7 cells (Fig. 6A). This effect is consistent with the reported activity of 4-OHT as an ERα agonist in the context of SP1 and AP-1-regulated genes (Schultz et al., 2005), indicating that SP1 is required in promoting *ADGRG7* promoter activation. ICI-182, 780 is known as a potent ERα antagonist that promotes ERα degradation and abolishes its transcriptional competence to E2 in responsive cells. We observed that increasing concentrations of ICI-182, 780 completely abolished the response of the *ADGRG7* promoter to E2, thus suggesting a direct role of ERα (Fig. 6B). We also tested the effect of ICI-182, 780 treatments on the SP1 mutant. Contrary to the inhibitory effects observed with ICI-182, 780, there was no response with the SP1 mutant (Fig. 6C).

We also tested the effects of ICI-182, 780 and 4-OHT on ADGRG7 protein levels (Fig. 6D,E). Both antagonists downregulated the E2 induced ADGRG7 protein levels.

Based on the above results, both SP1 and ERα are responsible for the regulation of *ADGRG7*, and the mutation of SP1 prevents the response *of ADGRG7* to the ERα antagonists.

## DISCUSSION

**Fig. 7.**
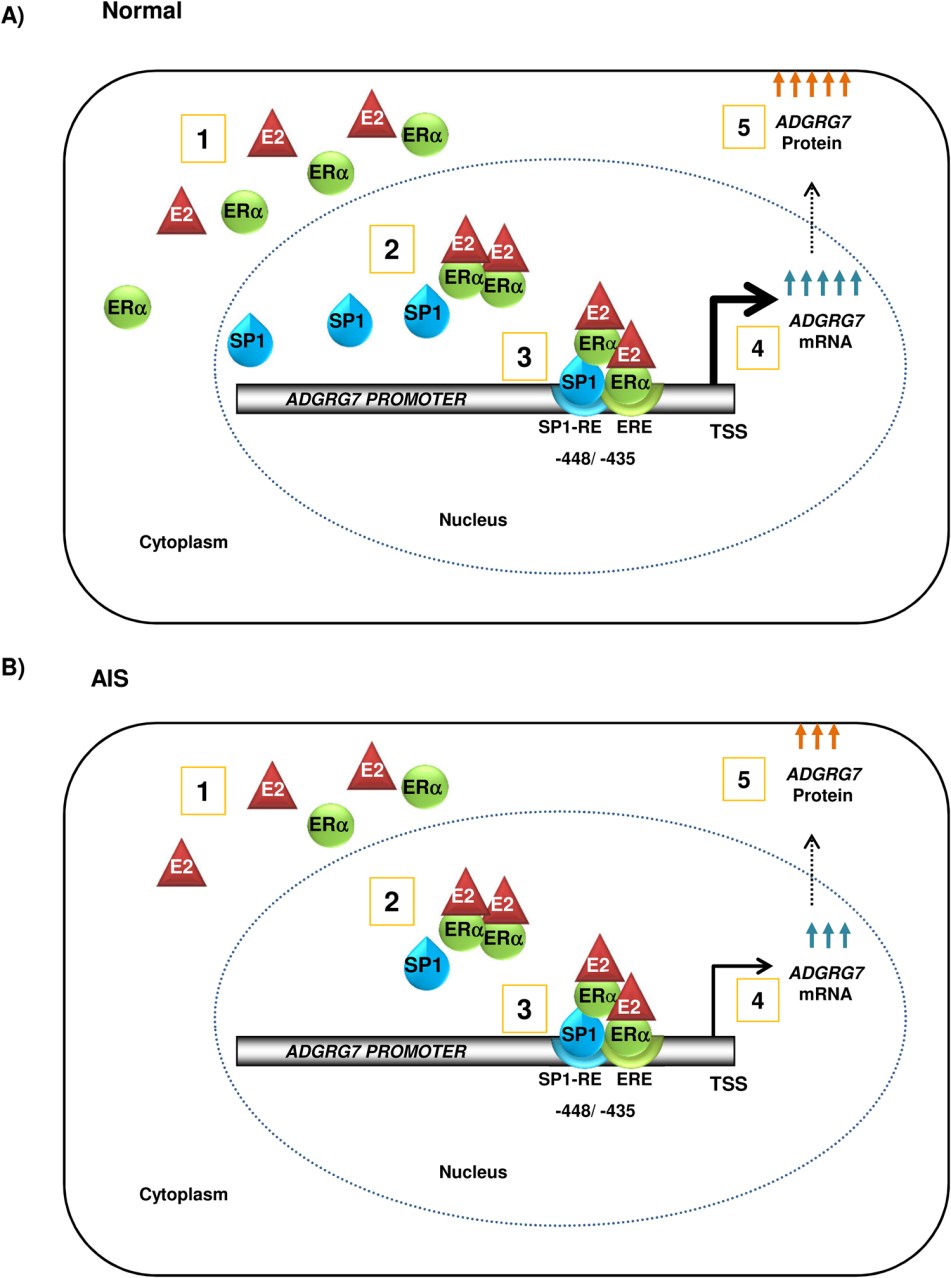
**Schematic representation of the effects of E2 in normal and AIS cells.** The transcriptional regulation of ADGRG7 is driven by both the genomic signaling (direct and indirect) of estrogen in conjunction with SP1. Through promoter studies, we found that estrogen acts mostly through ERα to induce the expression of ADGRG7 in cells through the distal promoter ERE half-site along with the SP1 binding site or response element (SP1-RE). In AIS cells, there is decreased expression of ERα and SP1. The induction of the transcription of ADGRG7 by E2 is lower in AIS cells from scoliotic patients compared to the cells from normal control individuals.

In the present study, we demonstrate *ADGRG7* regulation by E2. This is the first report demonstrating that ER signaling regulates *ADGRG7* expression and activity. Additionally, we showed the mechanism of regulation of *ADGRG7* by E2 represented in Fig. 7. Our results indicate that the activity of E2 on the *ADGRG7* promoter was dependent on SP1-binding sites in the promoter (Fig. 4B). The latter acts as critical regulatory *cis*-acting element mediating the activation of ADGRG7 transcription by ERα. ERα, but not ERβ, induced *ADGRG7* promoter upregulation. ERβ is thought to have weaker activity than ERα, although in some studies it was shown that the ERβ a better activator than ER-α on an ERE reporter (Fournier et al., 2001). Also we found that in MCF7, the E2 antagonists 4-OHT and ICI-182, 780 inhibited E2-stimulated promoter activity as well as protein levels (Fig. 6), suggesting that also ERα is important in the *ADGRG7* regulation by E2.

Other than the classical pathways of estrogen signaling, ERα can mediate E2 regulation through tethered interactions with SP1 protein to regulate genes and this occurs by the binding of SP1×ERE or SP1×ERE half-site (1/2) motifs where both ERα and SP1 bind DNA elements (Safe, 2001). As it seems to be the case for *ADGRG7*, there is activation through SP1(5)×ERE1/2 that is located in the Δ67 bp fragment of the *ADGRG7* promoter. Several examples exist in literature for genes devoid of ERE having SP1 and ERE1/2 sites. The E2-responsive SP1(5)×ERE(1/2) motif regulation mechanism of *ADGRG*7 by E2 identified in this study is also shared with other E2-regulated genes, such as cathepsin D (Krishnan et al., 1994, 1995), transforming growth factor alpha (TGFα) (Vyhlidal et al., 2000), heat shock protein 27 (Porter et al., 1996) and the progesterone receptor (Petz and Nardulli, 2000). Cathepsin D doesn't contain a classical palindromic ERE. The promoter region (between −199 and −165) has an ERE1/2 and an SP1 binding that are mediating upregulatory E2 effects on cathepsin D expression (Krishnan et al., 1994, 1995). Similarly, TGFα promoter required SP1(30)×ERE½ that had been characterized in the cathepsin D, progesterone receptor and heat shock protein 27 gene promoters. Many other examples of the SP1×ERE1/2 in promoters of several E2-responsive genes, include cyclin D1, c-fos, retinoic acid receptor α1, E2F1, adenosine deaminase, insulin-like growth factor binding protein 4, creatine kinase B and B-cell lymphoma 2 (bcl-2) (Khan et al., 2003).

The regulation of GPCRs by E2 has been described for several receptors. For instance, E2 can upregulate or downregulate the expression of mRNAs of several genes involved in lipid metabolism, transcription, and steroid metabolism that have a role in the control of reproductive behavior (Snyder et al., 2009). E2 also increases the expression of mRNA and protein levels of oxytocin receptor in human placenta cells (Kim et al., 2017).

No SP1 mutation association with scoliosis has yet been reported. However, mutation in the binding site of SP1 transcription factor (G–>T mutation) in the collagen type I alpha 1 gene (COLIA1) is a putative marker for low bone mineral density (Grant et al., 1996). Based on these findings, it would be interesting to screen for SP1 binding site mutations in the *ADGRG7* gene in osteopenic scoliotic patients.

Interestingly, at low E2 dose, the upregulation of *ADGRG7* by E2 in normal osteoblasts was higher than in scoliotic osteoblasts probably due to lower protein levels of SP1 and ERα (Fig. S2). An E2 resistance mechanism in scoliosis was previously reported in humans with a mutation of the *ERα* gene. In exon 2 of *ERα*, a cytosine-to-thymine transition at codon 157 of both alleles resulted in a premature stop codon (Smith et al., 1994). The major phenotypic manifestations of this mutation were a severely under mineralized skeleton with biochemical evidence of increased bone resorption, evidence of continued slow linear growth, markedly delayed skeletal maturation and osteoporosis. E2 resistance impacts bone turnover (Quaynor et al., 2013) that could be connected to the molecular mechanisms underlying AIS. Since E2 is important for bone growth and mineralization, this could explain the low mineralization and osteopenia that was observed in patients with AIS (Li et al., 2008). In a morpholino zebrafish model, ADGRG7 variant affected bone development resulting in very low calcification suggesting a role for this gene in bone formation and bone mineral density regulation in AIS.

ADGRG6 is also connected with the reduced body mass index and its activity/function seems to be correlated with osteoblast metabolism and bone calcification (Kou et al., 2013) (both altered in idiopathic scoliosis). Recently, a rare variant of cadherin EGF LAG seven pass G-type receptor 2 (CELSR2) was co-segregating with scoliosis in Swedish-Danish patients (Einarsdottir et al., 2017). CELSR2 is an adhesion GPCR and plays a role in neuronal system development along with other physiological processes. The missense mutation in CELSR2 is located within the highly conserved GAIN domain. The consequences of this mutation on the structure of protein are not expected to be major; however, structure predictions of the mutant (mut) CELSR2 indicate that it is located in close proximity to the H2355-T2357 autoproteolysis cut site. Homozygote loss of function mutations in *ADGRG6* were found to be associated with lethal arthrogryposis. Scoliosis occurrence in a patient with arthrogryposis was reported. The mutation (c. 2306T>A; p. Val769Glu) was located in the GAIN domain of ADGRG6, and results in a reduced but not a complete elimination of autoproteolytic activity (Ravenscroft et al., 2015). The GAIN domain is highly conserved through evolution and it has a function in properly activating the receptor (Arac et al., 2012b). Interestingly, the mutation in *ADGRG7* (1274A>G) is also located in the GAIN domain. Since the mutation in *ADGRG7* is heterozygote, it doesn't significantly affect the function of ADGRG7 protein, and it is possible that homozygote mutations would abolish ADGRG7 activity resulting in a more severe phenotype of AIS. Future studies are thus needed to determine how *ADGRG7* mutation could disrupt the auto-proteolytic mechanism of the GAIN domain in ADGRG7.

## CONCLUSION

This study reports the mechanism of *ADGRG7* regulation by E2, with differential response of normal and scoliotic osteoblasts, and suggests that *ADGRG7* is a contributor risk gene in AIS. The effect of E2 pointed out the disruption of the auto-proteolytic mechanism of the GAIN domain in the *ADGRG7*. The differential response of normal and scoliotic osteoblasts to E2 suggests that molecular mechanisms and pathways associated with AIS (progression and/or the onset) could be associated with the rise in sex hormones (including E2). The functional consequences of *ADGRG7* upregulation as well as the gene variants of the adhesion subfamily of G-protein coupled receptors that are possibly contributing factors in the pathogenesis of AIS during the pubertal growth spurt merits further *in vivo* examination.

## MATERIALS AND METHODS

### In-silico analysis of gene expression

The expression profiles of target genes in normal and cancer samples generated from the affymetrix platform 133plus2 were downloaded using GENT (gene expression across normal and tumor) software (Shin et al., 2011). GENT (http://medicalgenome.kribb.re.kr/GENT/reference.php) uses datasets created by the Affymetrix platforms (U133A and U133plus2). The data of normal tissues was then analyzed using one-way analysis of variance performed.

### Cell culture and treatments

The human hepatocellular carcinoma cell line Huh7 cells was a kind gift from Dr M. Santo and cultured as previously published (Bagu and Santos, 2011). Human breast cancer MCF-7 cells were purchased from American Type Culture Collection (American Type Culture Collection). MCF-7 cells were cultured in Dulbecco's modified Eagle's medium (Wisent) supplemented with 10% fetal bovine serum (Wisent) and 1% Penicillin-streptomycin antibiotic. Primary human osteoblasts were isolated and cultured as described (Fendri et al., 2013). For estrogen assays, cells were cultured in phenol red-free DMEM (Wisent) supplemented with 10% charcoal-stripped fetal bovine serum (CS-FBS) for 3 days. The next day, the cells were treated with ethanol (vehicle) or 10^−7^ M E2 for 24 h. E2 and 4-hydroxy-tamoxifen (4-OHT) were purchased from Sigma-Aldrich, ICI-182, 780 was from Tocris Bioscience. E2, along with its inhibitors ICI-182, 780 and 4-OHT, were reconstituted in 100% ethanol as stock solutions of 2×10^−2^ M and stored at −20°C as indicated by the manufacturer.

### Patients

For AIS patients, at the surgery, bone biopsies were collected from the vertebrae of the spine (varying from T3 to L4 according to the surgical procedure performed). For AIS patients, the following criteria were used: a diagnosis of AIS documented by a Cobb angle >30 and a therapeutic indication for corrective surgery and females aged between the 10 and 20 years old. For the normal control osteoblasts, tissue was collected from non-scoliotic females aged between 10 and 20 years old, who were operated on because of a traumatic spinal condition. For scoliotic cells, tissue was collected from female patients with mean age of 14.80±1.83 and menarche mean age of 13.2±1.53. The tissues were collected with consent following approval by the Institutional Ethics Committee Board of CHU Sainte-Justine Hospital Montreal, Canada.

### RNA extraction and qPCR analysis

Total RNA was isolated using TRizol as recommended by the manufacturer (Invitrogen Canada). RNA (2 μg) was used as a template to synthesize the first strand-cDNA using Superscript reverse transcriptase (Bio-Rad). Total RNA was derived from cells and the cDNA was synthesized using iScript reverse transcriptase (Bio-rad). Quantification of gene expression was performed by 7900HT Fast Real-Time PCR System (Applied Biosystems Stratagene) with iQ™ SYBR® Green Supermix (Bio-rad). The oligonucleotides used to amplify *GAPDH* and *ADGRG7* are listed in Table S4. The generated PCR products were confirmed by agarose gel electrophoresis and sequencing (McGill University and Génome Québec Innovation Centre, Montreal QC). Gene expression levels were normalized to *GADPH* expression and expressed as fold change compared to vehicle-treated cells derived from at least three separate experiments. All treatments were normalized to the control value, which was 1.

### In-silico analysis of *ADGRG7* promoter

Multiple bio-informatics tools [Evolutionary Conserved Regions (ECR)-Browser, http://ecrbrowser.dcode.org] determined putative regulatory elements (RE) in the *ADGRG7* promoter for ERα and SP1.

### *ADGRG7* promoter constructs and mutagenesis

A 2204 bp fragment corresponding to the proximal promoter (−2259 to +55) of *ADGRG7* was generated by PCR (primers listed in Table S1) from genomic DNA isolated from Huh7 cells. The fragment was then cloned into a pGL3 basic luciferase reporter plasmid vector (Promega) to generate (−1285/+55) promoter construct. A series of deletion constructs (−474/+55; −309/+55 and −283/+55 and −44/+55) were generated by PCR amplification using the *ADGRG7* plasmid −1285/+55 as a template and primers are listed in Table S1.

Mutated SP1 construct was obtained by site-directed mutagenesis using the QuikChange II XL mutation procedure (Stratagene) with respective primer pairs (Table S2) according to manufacturer’s instructions. The constructs were validated by automated sequencing (McGill University and Genome Quebec Innovation Center). Expression vectors coding for GFP-flagged human estrogen receptor ERα (pEGFP-hERα) and ERβ (pEGFP-hERβ) were obtained from Addgene (#28230 and #28237).

### Protein lysate preparation and western blotting

Whole cell protein lysates were prepared from cultured cells using RIPA buffer (Pierce, Thermo-Fisher Scientific) (25 mM TrisHCl pH 7.6, 150 mM NaCl, 1% NP-40, 1% sodium deoxycholate, 0.1% SDS,) supplemented with protease and phosphatase inhibitors (Roche Diagnostics). Samples were resolved by 10% SDS-PAGE and transferred to nitrocellulose membranes (Millipore). Membranes were blocked with (20% skim milk) and probed with primary antibody (1:250) against ADGRG7 (Thermo-Fisher Scientific), SP1 (Abcam), ERα and β-actin (Santa Cruz Biotechnology). After being washed with phosphate-buffered saline containing 0.05% Tween 20, membranes were probed with a horseradish peroxidase-conjugated secondary antibody (1:10,000; Thermo-Fisher Scientific) for 1 h. Signals were visualized with a chemiluminescent substrate (ECL Plus western blotting detection system, Amersham Biosciences).

### Transfection and luciferase reporter assay

Transfections of Huh7 hepatoma cells and MCF7 were performed in 24-well plates using Lipofectamine™ 2000 (Invitrogen) as recommended by the manufacturer. Briefly, on the day before transfection, approximately 9×10^4^ cells (Huh7 and MCF7) were seeded per well in a 24-well plate in phenol red-free DMEM supplemented with 10% CS-FBS. Cells were co-transfected with 990 ng/ well of the different *ADGRG7* promoter constructs (−2259/+55; −1285/+55−474/+55; −309/+55 and −283/+55 and −44/+55) along with 10 ng of phRL-TK vector (Renilla Luciferase; Promega) according to manufacturer’s protocol. The total DNA per well was kept at 750 ng/well in the 24-well plates by co-transfecting with the empty expression vector pCDNA3. When evaluating the effect of ERα on *ADGRG7* in the presence or absence of E2, ERα negative Huh7 cells were co-transfected with different *ADGRG7* promoter constructs along with either the expression vector encoding the full length human ERα protein (250 ng/well) or the empty pCDNA-3 vector (Invitrogen). After a 24 h transfection, the cells were washed and treated for 24 h with fresh phenol red-free DMEM supplemented with 1% CS-FBS containing E2, 4-OHT or ICI-182, 780 only dissolved in ethanol, or ethanol alone as a vehicle control. The cells were harvested, and subsequently luciferase activity was determined with a luciferase assay system (Promega) according to the manufacturer's directions. Luciferase activity was normalized to the activity of co-transfected Renilla luciferase as an internal control for transfection efficiency. In order to evaluate the basal luciferase activity for each construct, controls for each full-length promoter construct were co-transfected with an empty pCDNA-3 vector (Invitrogen) and then cultured in the vehicle. In all experiments, data reported represents the average of at least three experiments, done in triplicates.

### ChIP

ChIP was performed as described (Edjekouane et al., 2016). Briefly, MCF7-ERα cells were cultured in phenol red-free medium and 10% charcoal stripped FBS then treated with 10^−7^ M E2 or vehicle for 1 h. After fixation with 2% formaldehyde, cells were lysed and the precleared chromatin supernatants were immunoprecipitated with the respective antibodies specific anti-ERα (Santa-Cruz, cat# sc-542) and anti-SP1 (Abcam, cat# ab13370) or nonspecific IgG at 4°C. Bound DNA was purified with phenol/chloroform and used as a template for subsequent amplification using primers (see Table S3) that encompass respective specific binding elements within the proximal ADGRG7 promoter region. Fold enrichment values were calculated using the Ct value of each ChIP sample compared to the Ct value of Input DNA. The qPCR products were migrated on 2% agarose gel to confirm the correct product size.

### Fluorescence microscopy

Osteoblasts from normal and AIS patients were seeded on Labtek (NUNC, Thermo-Fisher Scientific) and cultured overnight. On the second day, cells were treated with 10^−7^ M E2 for 24 h. On the third day, cells were fixed in 70% ethanol/0.1% triton on ice for 30 min. Cells were then washed with PBS and permeabilized with 0.1% Triton in PBS for 15 min. Cells were washed once with 0.5% BSA in PBS/Triton (PBT), blocked with 2% BSA in PBT for 45 min, and incubated with the anti-ADGRG7 antibody (Thermo-Fisher Scientific) at (1/200) for 1 h with gentle shaking at room temperature (RT). Cells were then incubated with Alexa Flour 488 goat anti-rabbit (Life Technologies USA, cat# A11008) for 1 h. Cells were mounted and stained for nucleus at the same time using Prolong Gold antifade reagent with DAPI (Life Technologies). Immunostaining was examined at magnification 40×.

### Statistical analysis

The data collected on the expression of ADGRG7 (mRNA and protein), and promoter activity in different cells types (Huh7, MCF7, Osteoblasts) were analyzed for effects of treatments [estradiol, estradiol receptor inhibitors (ICI 182,780 or 4-OHT), vehicle] (over time) by a one-way analysis of variance (ANOVA, Sigma Stat for Windows^®^, version 1.0; Jadel Corporation). In the case of Osteoblasts, comparisons were made of normal versus AIS. If the main effects were significant, multiple comparisons were done using Fisher's LSD method or the Newman-Keuls test for post-ANOVA multiple comparisons (*P*<0.05). Statistically different means from the controls were indicated by either *P<0.05 or **P<0.01. Different superscripts were used to indicate paired means for each treatment that were statistically different *a*, *b*, *c*, *x*, *y*, *z* (*P*<0.05). For variables with the same letter, the difference is not statistically significant. Likewise, for variables with a different letter, the difference is statistically significant (*P*<0.05) (Assaad et al., 2015). Data are reported as mean±S.E.M. Pearson's correlations were done to evaluate the consistence of the data and the relationship across gene expression profiles. For luciferase and qPCR experiments, data are representative of at least three independent experiments in triplicates. Student’s *t*-test was used and *P*<0.05 was considered statistically significant.

## Supplementary Material

Supplementary information
