# Supplementary material for: The 17β-estradiol induced upregulation of the adhesion G-protein coupled receptor (ADGRG7) is modulated by ESRα and SP1 complex
Source: Biol Open. 2018 Dec 31;8(1):bio037390. doi: 10.1242/bio.037390 (PMC6361214; doi:10.1242/bio.037390)
Supplement: Supplementary information [file biolopen-8-037390-s1.pdf]

A

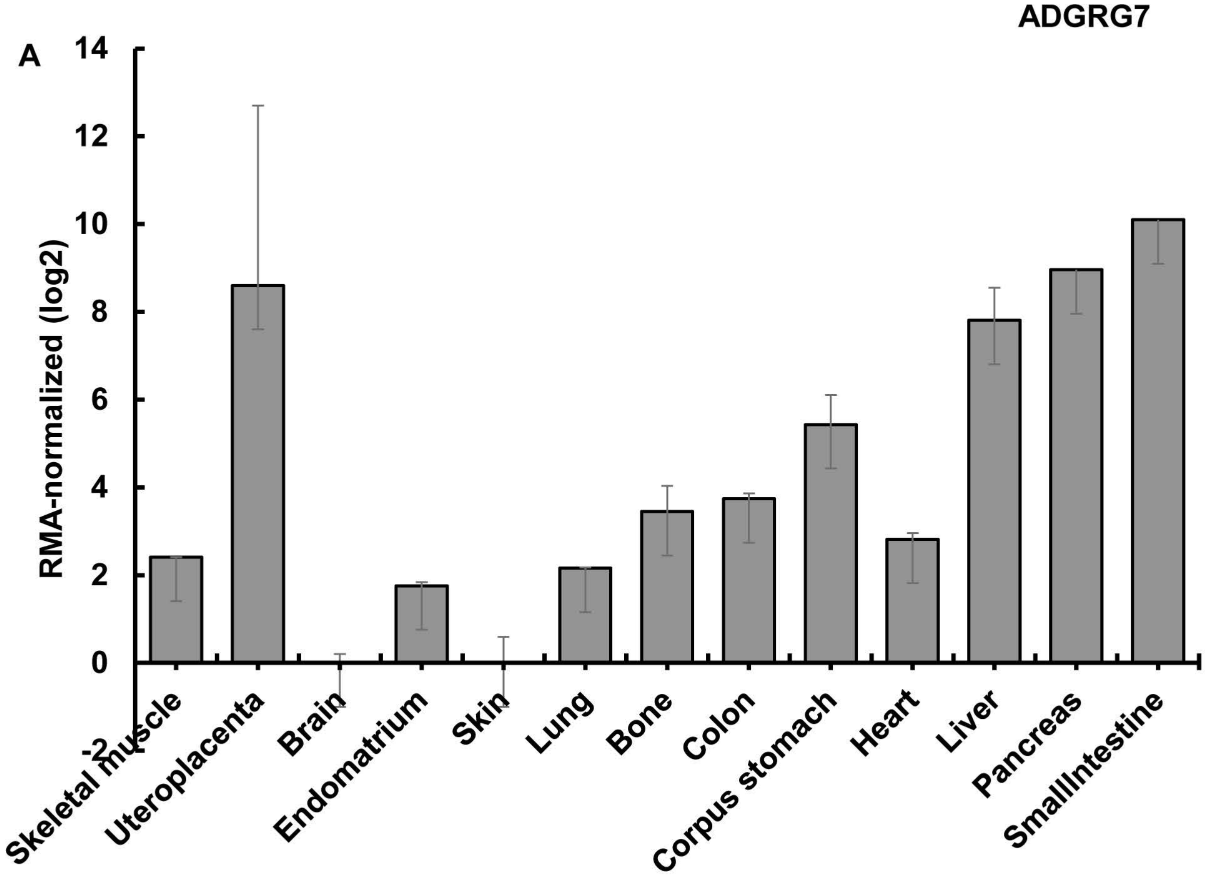

B

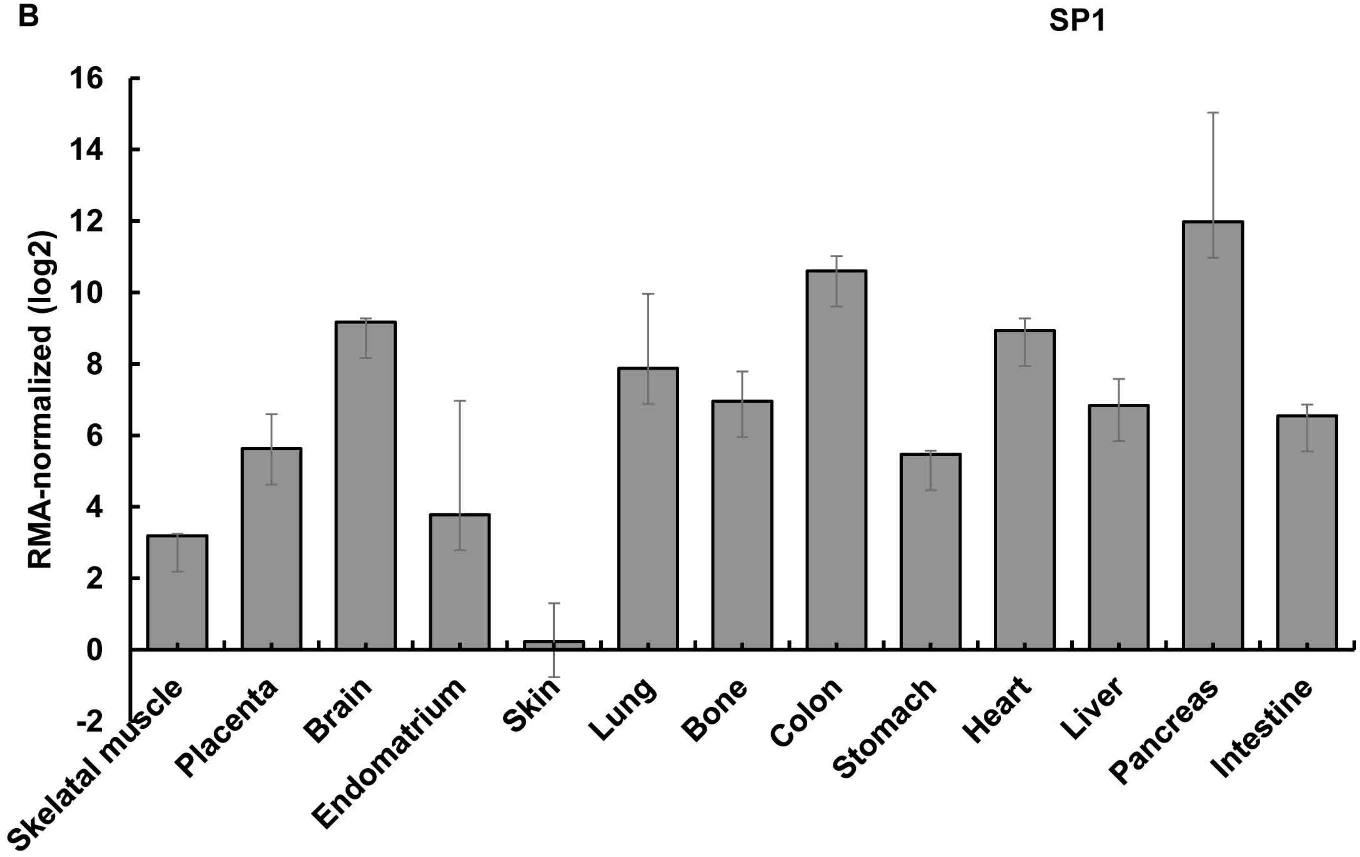

Biology Open • Supplementary information

Figure S1. ADGRG7 and SP1 expression in human tissues. ADGRG7 (A) and SP1 (B) microarray expression in normal human tissues were extracted from GEO database available from NCBI. The profiles of the target genes in human tissues were collected from different studies and the mean transcript levels determined. The data is presented as mean ± SD.

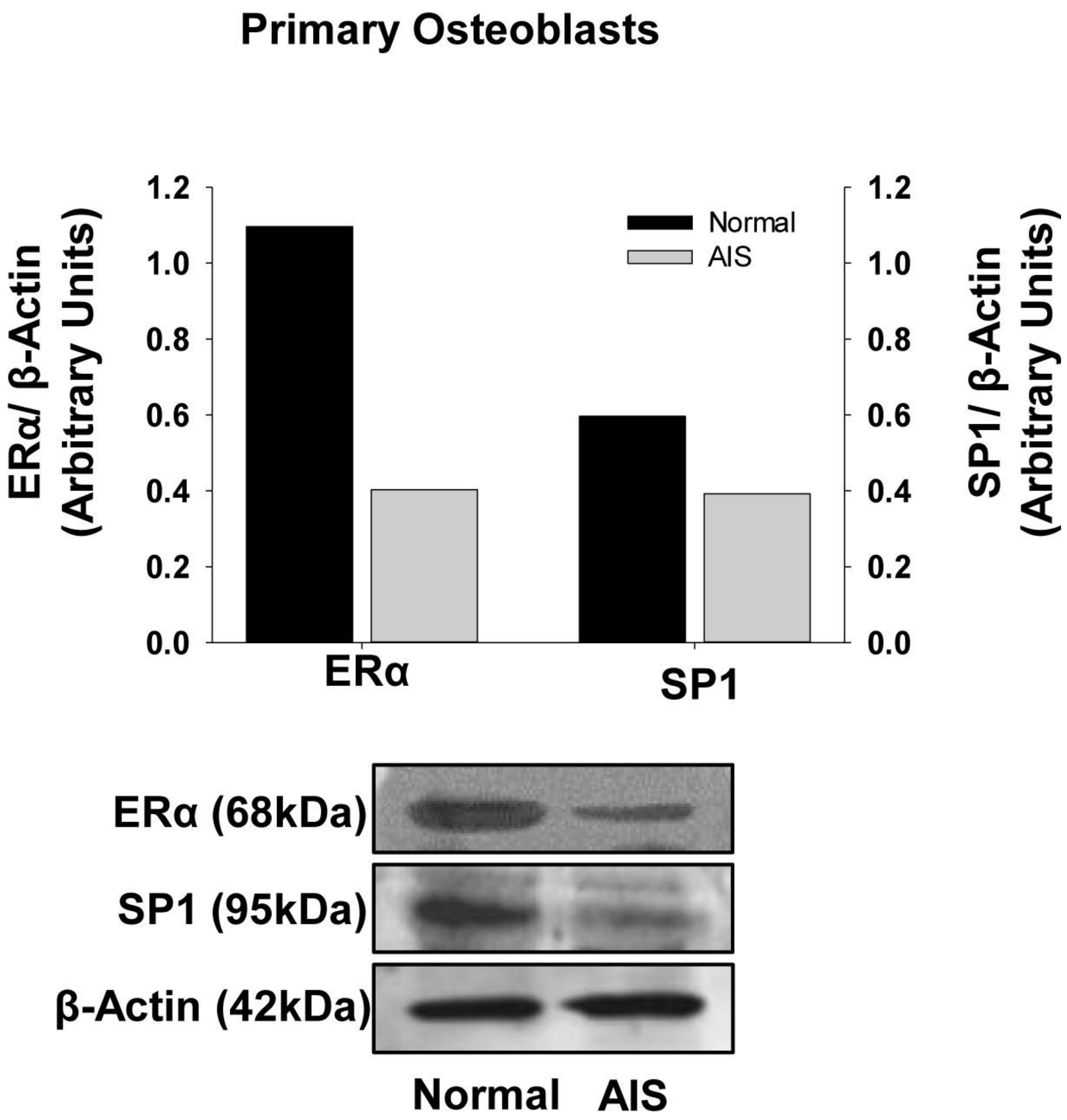

Figure S2. Western blot analysis of ERα and SP1 in NOB and AIS cells. Total cell lysates were isolated. Detections of ERα, SP1 and β-actin were performed by immunoblotting as described in Materials and Methods. Protein quantification was done using Image-J.

Biology Open • Supplementary information

Table S1. List of primers used to clone the ADGRG7 promoter full length and deletion constructs. The primer direction was represented as F and R represent the forward and reverse directions respectively. The first column from left indicting the amplicon length generated with the primer pairs in the raw and the second column indicating the beginning and end of the primer sequence with respect to the transcriptional start site.

**Length (Bp)**	**Primer Position**	**Sequence**
1397 (1/2)	-1318/-1291	F: 5’-GTATGTGTTTTGGGCACATTTAAGGTA-3’
	+82/ +112	R:5’TAGATCGCAGATCTCGAGGCTAATCCAGTAGGATGCAGTTTGAATC-3’
421 (3/2)	-309/ -285	F: 5’-GAAATGCTAGCGCTAAAAACCATGTGTGGTGATT-3’
	+82/ +112	R:5’-TAGATCGCAGATCTCGAGGCTAATCCAGTAGGATGCAGTTTGAATC-3’
283 (4/ 2)	-171/ -147	F: 5’-ATACAGCTAGCCGAGCCATAGGTGACTACTGGC-3’
	+82/ +112	R:5’TAGATCGCAGATCTCGAGGCTAATCCAGTAGGATGCAGTTTGAATC-3’
156 (5/2)	-44/ -22	F: 5’-CTTGTGGCTAGCCCCTCCCCTTCTTCTTTATTGCC-3’
	+82/ +112	R:5’TAGATCGCAGATCTCGAGGCTAATCCAGTAGGATGCAGTTTGAATC-3’

**Length (Bp)**	**Primer Position**	**Sequence**
Δ974 (3/4)	-2259/ -2235	F: 5’-AACCATAGAGCTCCTGTGTAGAATAATACCAGTTT-3’
	-1283/ -1257	R: 5’-ACCTAACCTGCTGAACATCCTTGCTTA-3’
Δ101 (10/11)	-385/ -359	F: 5’-GCTTTAGTGCTAGCCAAGACCCAGCCTGTGCCAGTATGCAC-3’
	-309/-285	R: 5’-CAATCACCACACATGGTTTTAGCGCTAGCATTTC-3’
Δ60	-676/ -651	F: 5’-CTTCTGAGGACTCTCTCTTGTAGATG-3’
	-451/ -419	R: 5’-GCTAGCGCTATGGGCTGAATTGTG-3’

Table S2. The forward and reverse primers that were used to mutate the SP1 response element in the ADGRG7 promoter construct (-474/+112). The second column from the left indicates the beginning and end of the primer sequence with respect to the transcriptional start site.

**Application**	**Position relative to TSS**	**Sequence**
SP1 Mutant	-463 / -421	Forward: 5'-CTCCAACATATGAATGTTCCATGGGACACAATTCAGCCCATAG-3'
	-463 / -421	Reverse: 5'-CTATGGGCTGAATTGTGTCCCATGGAACATTCATATGTTGGAG-3'

Biology Open • Supplementary information

Table S3. List of primers used for Chromatin Immunoprecipitation (ChIP) of the SP1 and ESRα response element on the ADGRG7 promoter. The second column from the left indicating the beginning and end of the primer sequence with respect to the transcriptional start site.

**Use**	**Position relative to TTS**	**Sequence**
CHIP	SP1	Forward: 5'- GACCACCCTAACAAACTCG- 3
		Reverse: 5'- GATAACCCACCTGCTATGG-3'
	ESRα	Forward: 5'- CCAACTTGGATTCTCCAGCTCC-3'
		Reverse: 5'-CAACAGGTCACCTCAAAACTTAGGG-3'

Biology Open • Supplementary information

Table S4. Primers used for qPCR.

Application	Description	Sequence
QPCR	GAPDH	Forward : 5'AGGAGTAAGACCCCTGGACC3'
		Reverse : 5' GGAGATTCAGTGTGGTGGGG3'
	ADGRG7	Forward 5'TGAAAGCAGAGTATGCACCTT3'
		Reverse : 5'TCCTCCCCTCAGTGATCTGT3'
